# Containment of COVID-19 in Ethiopia and implications for tuberculosis care and research

**DOI:** 10.1186/s40249-020-00753-9

**Published:** 2020-09-16

**Authors:** Hussen Mohammed, Lemessa Oljira, Kedir Teji Roba, Getnet Yimer, Abebaw Fekadu, Tsegahun Manyazewal

**Affiliations:** 1 Addis Ababa University, College of Health Sciences, Center for Innovative Drug Development and Therapeutic Trials for Africa (CDT-Africa), P.O Box 9086, Addis Ababa, Ethiopia; 2 Department of Public Health, College of Medicine and Health Sciences, Dire Dawa University, Dire Dawa, Ethiopia; 3 School of Public Health, College of Health and Medical Sciences, Haramaya University, Harar, Ethiopia; 4 School of Nursing and Midwifery, College of Health and Medical Sciences, Haramaya University, Harar, Ethiopia; 5 Ohio State Global One Health Initiative, Office of International Affairs, The Ohio State University, Addis Ababa, Ethiopia; 6 Global Health and Infection Department, Brighton and Sussex Medical School, Brighton, UK

**Keywords:** COVID-19, Coronavirus, Public health, Tuberculosis, Containment, Ethiopia

## Abstract

**Background:**

The coronavirus disease 2019 (COVID-19) has emerged as a global health and economic security threat with staggering cumulative incidence worldwide. Given the severity of projections, hospitals across the globe are creating additional critical care surge capacity and limiting patient routine access to care for other diseases like tuberculosis (TB). The outbreak fuels panic in sub-Saharan Africa where the healthcare system is fragile in withstanding the disease. Here, we looked over the COVID-19 containment measures in Ethiopia in context from reliable sources and put forth recommendations that leverage the health system response to COVID-19 and TB.

**Main text:**

Ethiopia shares a major proportion of the global burden of infectious diseases, while the patterns of COVID-19 are still at an earlier stage of the epidemiology curve. The Ethiopian government exerted tremendous efforts to curb the disease. It limited public gatherings, ordered school closures, directed high-risk civil servants to work from home, and closed borders. It suspended flights to 120 countries and restricted mass transports. It declared a five-month national state of emergency and granted a pardon for 20 402 prisoners. It officially postponed parliamentary and presidential elections. It launched the ‘PM Abiy-Jack Ma initiative’, which supports African countries with COVID-19 diagnostics and infection prevention and control commodities. It expanded its COVID-19 testing capacity to 38 countrywide laboratories. Many institutions are made available to provide clinical care and quarantine. However, the outbreak still has the potential for greater loss of life in Ethiopia if the community is unable to shape the regular behavioral and sociocultural norms that would facilitate the spread of the disease. The government needs to keep cautious that irregular migrants would fuel the disease. A robust testing capacity is needed to figure out the actual status of the disease. The pandemic has reduced TB care and research activities significantly and these need due attention.

**Conclusions:**

Ethiopia took several steps to detect, manage, and control COVID-19. More efforts are needed to increase testing capacity and bring about behavioral changes in the community. The country needs to put in place alternative options to mitigate interruptions of essential healthcare services and scientific researches of significant impact.

## Background

The coronavirus disease 2019 (COVID-19) has emerged as a global health and economic security threat with staggering cumulative incidence worldwide. Declaring the disease as a global public health emergency, the World Health Organization (WHO) and different stakeholders have stepped up efforts to convince the world that the disease is a serious problem that needs resilient containment measures.

COVID-19 fuels panic in sub-Saharan Africa where the healthcare system is fragile in withstanding the disease. Governments in the continent responded swiftly in the early days of the pandemic, while there are concerns as some countries are experiencing a sharp rise in confirmed cases and countries have limited capacity for testing to early identify cases [1]. As of 25 June 2020, there have been 9 473 214 confirmed cases and 484 249 deaths (case fatality rate, CFR = 5.1%) reported worldwide. On the African continent, 258 752 COVID-19 cases and 5564 deaths (CFR = 2.2%) have been reported, accounted for 2.7% of cases and 1.1% of deaths worldwide [2].

Ethiopia, a country in sub-Saharan Africa, has a population of about 115 million, the second populous country in Africa and the twelfth globally. The median age is 19.5 years and 78.7% of the population is rural. It is also one of the list-resourced, with a per capita income of United States Dollars (USD) 850 in 2019 [3] and a human development index value of 0.47, at 173 out of 189 countries and territories [4]. Tuberculosis (TB), malaria, Human immunodeficiency virus infection and acquired immune deficiency syndrome (HIV/AIDS), and maternal mortality are the main health concerns in the country, and it is one of the countries with the highest burden of TB, TB/HIV, and multidrug-resistant TB (MDR-TB).

TB is yet the most killer infectious diseases in sub-Saharan Africa, and the fact that COVID-19 and TB have some similarities in clinical features is a potential risk for misdiagnosing the two. Though the incubation period for TB is longer with a slow onset, both diseases transmit through close contact and droplet particles and both affect the lung [5, 6]. The global scientific community is putting efforts to understand the COVID-19 crisis on TB care and treatment [7]. Healthcare systems, in general, are threatened by the rapidly increasing healthcare demand posed by the COVID-19 pandemic. Given the severity of projections, hospitals across the globe are creating additional critical care surge capacity and limiting patient routine access to care for other diseases like TB.

For countries in sub-Saharan Africa, the major health system shift into COVID-19, aggravated by poor health systems and ill-equipped healthcare facilities, is hampering the progress towards health target sets including the End TB [8]. When the COVID-19 pandemic had started in China, many African countries had a few laboratory testing capacities and logistic difficulties to track patients in their community [9]. In Ethiopia, TB program is problematic and the emergence of COVID-19 is assumed to worsen the situation [9–11]. Here, we looked over the COVID-19 containment measures in Ethiopia in context from reliable sources and put forth recommendations that leverage the health system response to COVID-19 and TB.

## Main text

### COVID-19 in Ethiopia

**Fig. 1:**
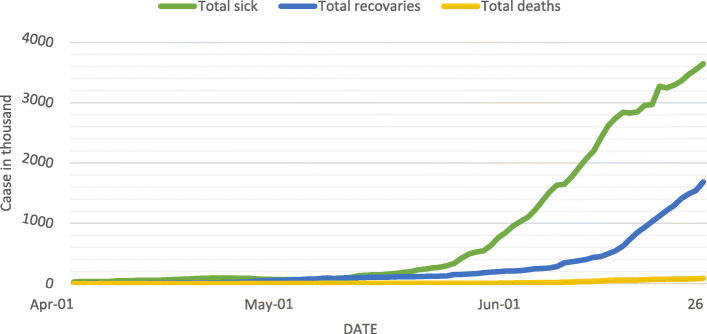
COVID-19 statistics of Ethiopia, 26 June 2020 [12]

Ethiopia confirmed its first case of COVID-19 on 13 March 2020, two days later the WHO declared a pandemic of the disease, and as of 26 June 2020, the country tested 237 464 suspects, of whom 5425 (2.3%) cases had been confirmed positive and of these, 89 (CFR = 1.6%) died and 1688 (31.1%) recovered (Fig. 1) [12]. The first case was a 48 years Japanese man who arrived in Ethiopia from Burkina Faso, and the second report was three cases, two Japanese and one Ethiopian, who had contact with the first Japanese person. Of those confirmed positive, 3325 (61.3%) were males.

Initially, before community transmission started, cases were largely imported and sourced from mandatory quarantines. Travel history was reported up to 2 June 2020. Of the total 1344 (24.8%) cases reported up to 2 June 2020, 408 (30.4%) were imported cases as they acquire the disease outside Ethiopia based on their travel history. Of these 408, 32 (7.8%) had a travel history to Dubai, 16 (4%) to Djibouti, and 9 (2.2%) to the United States, and they were in mandatory quarantines (Table 1).

**Table 1:** Travel history of COVID-19 confirmed cases in Ethiopia, 26 June 2020

Travel history	Number	%
Travel history not reported since 3 June 2020	4081	75.2
Travel history reported up to 2 June 2020	1344	24.8
No travel history up to 2 June 2020	936	69.6
Had travel history up to 2 June 2020	408	30.4
Dubai	32	7.8
Djibouti	16	3.9
USA	9	2.2
United kingdom	8	1.9
Punt land	8	1.9
Somalia	6	1.5
Sweden	4	1.0
Turkey	3	0.7
Congo Brazzaville	2	0.5
Belgium	2	0.5
Canada	2	0.5
Saudi Arabia	2	0.5
Australia	1	0.2
United Urabi Emirates	1	0.2
France	1	0.2
Germany	1	0.2
Israel	1	0.2
Japan	1	0.2
Lebanon	1	0.2
Burkina Faso	1	0.2
Had travel history to others up to 2 June 2020	306	75.0

Most of the cases, 5337 (98.4%), were Ethiopian nationals and this involved all nine National Regional States and two City Administrations of the country though the majority of cases, 3822 (71.6%), were reported from Addis Ababa, the capital city of Ethiopia (Table 2).

**Table 2:** COVID-19 cases by source country and national region

Nationality	Number of cases	Percentage
Ethiopian	5337	98.4
American	6	0.1
British	5	0.09
Chinese	5	0.09
Japanese	4	0.07
Eritrean	4	0.07
Indian	2	0.03
Equatorial	1	0.01
Canadian	1	0.01
Austrian	1	0.01
Libyan	1	0.01
Mauritian	1	0.01
Swedish	1	0.01
Israeli	1	0.01
Other nations	56	1.0
**Total**	**5425**	**100**
**Regions**		
Addis Ababa	3822	71.6
Somali	418	7.8
Amhara	297	5.5
Oromia	285	5.3
Tigray	204	3.8
^a^Other	399	7.0
**Total**	**5337**	**100**

^a^Other: Afar, SNNP, Benshangul Gumuz Gambela, Harari, Dire Dawa

### Public health interventions

The Federal Government of Ethiopia and its National Regional States and City Administrations took several progressive measures to combat the COVID-19 epidemic. (Table 3).

**Table 3:** Public health interventions to contain COVID-19 in Ethiopia

Core activities	Progressive measures taken to combat the COVID-19
Public gatherings suspended	On 16 March 2020**,** the government limited public gatherings including gatherings for religious practices, sporting events, and concerts, ordered school closures, ordered high-risk civil servants to work from home. The government preferred daily activities to continue but with containment measures
Transports restricted	Taxi and mass transport services were restricted to abide by new working time rules and to provide services with half of their load capacity. The nine Regional States and two City Administrations imposed travel restrictions
Flights suspended	Ethiopia suspended flights to 30 countries affected with the diseases on 23 March 2020 and this extended to more than 80 countries on 29 March 2020
Land borders closed	Ethiopia closed all land borders and deployed security forces on 23 March 2020
Pardon for prisoners	A total of 20 402 prisoners were granted pardon to prevent the spread of the disease in prison
Election postponed	Over COVID-19 fears, the Ethiopian government has officially postponed parliamentary and presidential elections which were supposed to be held on 29 August 2020
State of emergency declared	Considering the progressive rise of cases in the country, the government declared a five-month national state of emergency on 8 April 2020
Political parties participated	On 5 April 2020, the Prime Minister of the country Dr Abiy Ahmed officially met and discussed with leaders of competing political parties to discuss and reach consensus on the effect and containment of COVID-19
Religious leaders participated	Ethiopian Religious Council, which draws membership from various religions in the country, declared a one-month prayer program from 6 April to 5 May 2020 and this was televised live. Religious leaders had announced ahead for worshipers to avoid going to church and mosques but pray from home
Medias informed the public	Different national multimedia outlets and billboards massively disseminated facts and educational information to create awareness and deliver up-to-date information about COVID-19. Ethio-telecom uses cell-phone ring tones to remind people of the importance of hygiene measures
International collaborations harnessed	The country took steps in international collaborations to fighting the pandemic. Prime Minister of Ethiopia Dr Abiy Ahmed and the Chinese businessman Jack Ma and Ali Baba Foundation have initiated a PM Abiy-Jack Ma initiative to support African countries with COVID-19 diagnostics and infection prevention control commodities on 17 March 2020. The Ethiopian airlines deployed the COVID-19 supplied donated by the Jak Ma to African Union Member States and the African Union’s Center for Disease Control and Prevention provided technical guidelines. The commodities included millions of test kits, masks, and protective suits
Regular information dissemination	The Ethiopian Federal Ministry of Health and its technical arm, the Ethiopian Public health Institute, established an active surveillance mechanism as per the WHO recommendation to regularly check the status of the disease in the population and disseminate the information. The number of tests performed, cases confirmed, and cases recovered have been reported each day by Dr Lia Tadesse, State Minister of the Federal Ministry of Health. This has been the most reliable information

Despite all these important public health containment measures, the outbreak still has the potential for greater loss of life in Ethiopia if the community is unable to shape the regular behavioral and sociocultural norms that would facilitate the spread of the disease. Many Ethiopians live in crowded conditions [13] and this would facilitate the spread of the disease.

### Diagnostic interventions

At the initial phase of the COVID-19 containment, Ethiopia had only one federal-level laboratory at the Ethiopian Public Health Institute (EPHI) to conduct COVID-19 testing. This has improved significantly, with 38 national, regional, hospital, and private laboratories currently involved in the COVID-19 testing as of 26 June 2020. The validation for laboratories has performed and provided by EPHI. The collection of samples from suspects and contacts, transporting, and testing have been followed COVID-19 standard techniques recommended by the WHO. The PM Abiy-Jack Ma initiative and the WHO had significant contributions in the assessment, training, and establishment of COVID-19 testing laboratories in the country. However, a robust testing capacity that would expedite large-scale community-level surveillance of the disease is needed to figure out the actual status of the disease and reshape the containment strategies.

### Care and treatment intervention

The initial readiness assessments conducted in Ethiopia by the WHO documented several gaps and weaknesses in intensive care capacity for COVID-19. Since then, the country took several steps to upgrade its clinical care to isolate promptly and provide optimized care for persons suspected or confirmed cases. A rigorous contact tracing, isolation, compulsory quarantine, and treatment procedures and facilities have been established. The health system is tracked into three: Track one with health facilities providing a full range of services only for COVID-19 patients; track two with health facilities providing COVID-19 as well as routine care services as they have greater infrastructure and capacity; and track three with health facilities continued routine care services. Thousands of healthcare providers received training on case investigation, contact tracing, laboratory diagnosis, clinical care and treatment. The government introduced life insurance coverage for COVID-19 healthcare workers. The Federal Ministry of Health (FMoH) has developed several national COVID-19 implementation guidelines and protocols.

Public and private facilities have been identified and prepared at all regional states and two city administrations. In addition to this, different individuals have been provided their hotels, colleges, and universities that have been serving as quarantine centers. Some public universities’ dormitories have been converted to quarantine centers to increase the capacity to over 50,000 beds. A capital city hall, Millennium hall, has been changed to a temporary hospital, which has 1040 beds for coronavirus patients, out of which 40 are intensive care unit (ICU) beds. It started receiving patients on 02 June 2020. Besides, Youth Sports Academy has prepared to receive 300 patients. Suspect identification, testing, and isolation, as well as care and contact tracing, have been performed as per the national comprehensive handbook prepared by FMoH with different consultant bodies.

However, the facilities need to strengthen their laboratory testing capacity as different laboratory tests are needed to follow up and patient discharge. Besides, there are isolation centers that have no COVID-19 testing capacity but have been referring samples to other sites that would delay case detections and hamper.

### COVID-19 implications on TB care

In Ethiopia, the COVID-19 pandemic has reduced the routine TB diagnosis, care, and treatment significantly. TB cases detection rate has reduced considerably and Directly Observed Therapy visits have been interrupted. Dire Dawa, one of the two chartered cities in Ethiopia, is among the major sits for our project entitled Translation research into policy and practice: Scaling up Evidence-Based Multiple focus Integrated Intensified TB Screening to End TB (EXIT-TB). From the end of March 2020 where the first COVID case was identified in Ethiopia, there have been significant reductions in TB case detection. According to the data that we got from the Dire Dawa Health Bureau, there were 110 TB cases in the period 1 April to 30 June 2020, which was about three times lower than cases detected in the previous reporting periods (Table 4).

**Table 4:** TB cases in Dere Dawa City Administration before and after the COVID-19 pandemic

	Before	Before	Initial	In elevation
	2019 July August September	2019 October November December	2020 January February March	2020 April May June
All Tuberculosis form cases	326	342	270	110
Treatment outcomes				
Cure	85	79	75	11
Treatment completed	194	147	92	67
Lost to follow up	2	4	3	5
Death	10	5	10	6
Failure	0	2	0	0
Not evaluated	13	10	12	12

We see a similar challenge in Addis Ababa where we have sites for both EXIT-TB study and the SELFTB Trial (Electronic pillbox-enabled self-administered therapy versus standard directly observed therapy for tuberculosis medication adherence and treatment outcomes in Ethiopia: a multicenter randomized controlled trial) [14]. The SELFTB Trial included 10 public health centers with the largest TB client load. The average quarterly cases in the sites [14] have been reduced by two-third in the COVID-19 period.

Some health facilities that have been providing TB care and treatment services have been committed as COVID-19 isolation and treatment centers. Human and material resources for TB have been shifted to COVID-19, which also affected the TB case finding and care. Health care workers are frightened for themselves and their family for providing the services without essential adequate personal protective equipment (PPE). In such cases, the smooth transition of patients’ follow-up to nearby health facilities with their full documentation is required through creating a system for these purposes between sending and receiving health facilities. Besides, for referral linked facilities, pre-informing to which health facility they will send their patients should be considered as early as possible. As TB symptoms and COVID-19 symptoms are overlapping, awareness creations should be continuously performed and reminded the health care workers to avoid missing more TB cases in Ethiopia where a third of TB cases have missed. Social stigma has been witnessed for patients co-infected with TB. Health education has the potential mitigating stigmatization [15, 16]. Thus, a unique health education platform that connects the two diseases is strongly needed.

People with TB are at higher risk of infection and deaths with COVID-19. Considering this, WHO recommended simultaneous testing for TB and COVID-19 [2]. As Ethiopia is among high TB burden countries, a system of routinely testing all TB cases for COVID-19 is important. The specimen of interest and diagnostic modality for the two diseases are quite distinct and this may call the country to mobilize additional resources and a different supply-chain system. At current times, the supply-chain for TB diagnostics is affected by COVID-19 restrictions and lockdown. Xpert MTB/RIF and realtime MTB and RIF/INH testing tools are shown to be useful diagnostic tools for COVID-19 as well. To sustain TB diagnosis, treatment and care, people-centered and community-based care and treatment need to be strengthened. To avoid interruption of TB care and minimize exposure of TB patients to COVID-19, a novel and verified intervention mechanism is needed.

In Table 5, we summarized the COVID-19 related critical issues and problems affecting TB care and treatment program in Ethiopia and potential solutions that would mitigate the challenges.

**Table 5:** Critical issues for TB program during COVID-19 pandemic and suggested solutions

Current situation and problems	Suggested solutions
Low infection prevention control	Increase provisions of personnel protective equipment for health care workers working at DOTS clinic, including N95 respirator, hand washing, use of gloves, decontaminations of surfaces regularly, use of ventilators, keeping the physical distance at the workplace, use safety of sample taking and transporting as per triple packing standards
Increased stigma against TB patients as the symptoms are overlapping with COVID-19	Health education should be strengthened for the public, TB patients, and health care workers; capacity for screening for both diseases during patient’ health facility visits should be strengthened
Incomplete TB service transfer to newly established sites for temporary services	When TB services are transferred to newly established facilities, it should compy the standard TB diagnosis, care, treatment, and prevention measures
Weak TB patients’ transfer to other health facilities	When transferring TB patients from one facility to another, patient preference should be considered and full information of the patient should be shared with the new facility
High interruption of laboratory services	Laboratory supply-chain system should be strengthened to balance the testing needs of TB and COVID-19
Weak contact tracing, surveillance, and monitoring	To minimize interruptions, facility-based contact tracing and surveillance could be switched to a home-based system through mHealth platforms and the role of community health workers should be considered to sustain the TB surveillance and monitoring
Lack of attention for TB care for a marginalized and vulnerable population, and people at COVID-19 quarantine and isolation centers	All COVID-19 confirmed or suspected people should have full access to TB diagnosis and treatment services as per the standard procedure
High fear and less benefit of healthcare workers at DOTS clinics	Healthcare workers at TB clinics need to get adequate training on COVID-19, mental health and psychosocial support, and incentives to maintain quality of and uninterrupted TB services
The potential use of health technologies to monitor TB treatment adherence is rarely explored	Studies that evaluate the effectiveness of TB digital adherence technologies in Ethiopia need to be conducted

*DOTS* Directly observed treatment, *COVID-19* Coronavirus disease 2019, *TB* Tuberculosis

### COVID-19 implications on TB research

The COVID-19 pandemic influenced many research activities across the globe; it affected data collection at the field and many scientists shifted their focus to COVID-19, including their laboratories. Researchers understood that research activities need to protect the participants’ and research staffs’ wellbeing. For these reasons, many researchers prefer to keep hold of their research in the COVID-19 outbreak.

We are currently implementing the EXIT-TB research project, which is funded by the European and Developing Countries Clinical Trials Partnership (EDCTP2) program under the European Union (CSA2016S-1608). A co-author of this study is implementing a clinical trial on TB with financial support from the U.S. National Institutes of Health (D43TW009127) [14]. COVID-19 has a significant impact on these studies. The studies require screening patients with TB and testing using different diagnostic modalities. Here, patients are not coming to healthcare facilities for a fear of COVID-19, and on the other side, TB services are marginally delivered and some sites stopped their routine services. For instance, one of the EXIT-TB study sites has been selected and prepared as a COVID-19 treatment center. Some patients on ant-TB treatment, their healthcare providers, and patients’ charts and have been transferred to nearby health facilities which are not in the study. TB services could sustain with this approach but significantly affect the researches. Research funding agencies do recognize the challenges and are looking for different mechanisms for the successful completion of such projects [17]. For instance, EDCTP vowed to accept a no-cost extension of research projects on top of all these difficulties, we believe that researchers' commitment should not be overwhelmed by COVID-19 and that they should look for options to complete started researches successfully or initiate new researches in the era of COVID-19.

Similar to Ethiopia, the links between TB and COVID-19 are most noticed in sub-Saharan Africa, where TB is the leading cause of death and the health system is weak to withstand the two diseases [18, 19]. Governments in sub-Saharan Africa African need to exert efforts on preventing the spread of the two diseases and continue the progress towards End TB strategy. WHO warned that Africa could be the next epicenter of COVID-19, with dual public health and economic crisis. It is crucial to keep an eye on the potential upsurge of COVID-19, but not at the cost of TB and other infectious diseases of global importance.

## Conclusions

Ethiopia took several steps to detect, manage, and control transmission of COVID-19. More efforts are needed to increase testing capacity and bring about behavioral changes in the community. The country needs to put in place alternative options to mitigate interruptions of essential healthcare services and scientific researches of significant impact.

## Data Availability

The dataset supporting the conclusions of this article is included within the article.

## Abbreviations

COVID-19: Coronavirus disease 2019
CFR: Case fatality rate
DOTS: Directly observed treatment, Short-course therapy
EPHI: Ethiopian Public Health Institute
EXIT-TB: Translation research into policy and practice: Scaling up Evidence-Based Multiple focus Integrated Intensified TB Screening to End TB
EDCTP: European and Developing Countries Clinical Trials Partnership
FMoH: Federal Ministry of Health
HCWs: Health care workers
SoF: State of emergency
SARS-CoV-2: Severe acute respiratory syndrome coronavirus 2
SELFTB Trial: Electronic pillbox-enabled self-administered therapy versus standard directly observed therapy for tuberculosis medication adherence and treatment outcomes in Ethiopia: a multicenter randomized controlled trial
TB: Tuberculosis
WHO: World Health Organization
